# Postoperative poor sleep quality and its associated factors among adult patients: A multicenter cross-sectional study^☆^

**DOI:** 10.1016/j.amsu.2022.103273

**Published:** 2022-01-31

**Authors:** Shimelis Seid Tegegne, Efrem Fenta Alemnew

**Affiliations:** Department of Anesthesia, College of Health Sciences, Debre Tabor University, Debre Tabor, Ethiopia

**Keywords:** Adult, Pittsburgh sleep quality index, Poor sleep quality, Postoperative prevalence, Sleep disorder

## Abstract

**Background:**

Sleep quality refers to a sense of being refreshed and rested after waking up from sleep. Postoperative patients were vulnerable to poor sleep quality due to having different contributing factors. The prevalence of poor sleep quality among postsurgical patients was left undisclosed in our study setting. Knowing the prevalence and contributing factors for poor sleep quality helps us to develop a strategic plan for prevention and management.

**Method and materials:**

A multicenter cross-sectional study design was conducted on 424 postsurgical patients who were selected by a systematic random sampling method. Data was collected using the Pittsburgh Sleep Quality Index by a face-to-face interview. Data analysis was done using SPSS version 25. For categorical data, a chi-square test was done. Bivariable and multivariable analyses were performed to determine whether each of the independent variables is associated with the outcome variable.

**Result:**

Based on this study result, the prevalence of poor sleep quality was 64.9%. Among the factors included in this study, variables which had an association with poor postoperative sleep quality were age range 25–54 years (AOR = 15.13), male gender (AOR = 4.81), educational level of secondary school (AOR = 6.29), patient income less than 2500 birr (AOR = 3.77), anxiety (AOR = 2.53), depression(AOR = 22.8), light exposure(AOR = 19.60), poor social support (AOR = 1.98), being emergency surgery (AOR = 2.46) and having a history of moderate to severe pain (AOR = 38.18, (95% CI).

**Conclusion:**

Poor sleep quality among adult post-surgical patients was significantly high in Amhara regional comprehensive referral hospitals. Therefore; Clinicians need to prioritize postoperative sleep quality assessment and needs to minimize factors inducing postoperative sleep disturbances.

## Highlights

• The prevalence of postoperative poor sleep quality was 64.9%.
• Pain is associated with poor sleep qualities.
• Postoperative sleep quality was poorly managed.

## 1 Background

Sleep is one of the vital physiologic processes that conserve body energy and help to restore activities [1]. Sleep disturbances can lead to mental illness, changes in body functions, and other health problems [2]. Different reports showed that more than 70 million people from developed countries suffered from sleep disturbances and it costs more than 150 billion dollars per year to treat the complications [3].

As a study showed, the prevalence of poor sleep quality was found to be from 33.8% to 67.3% in China [4,5]. In European countries, the prevalence of poor sleep quality was varied from 16.6% in Italy to 31.2% in Poland [6]. Whereas, the prevalence reaches 60.5% in Sub-Saharan Africa [7]. The sleep quality disturbance is caused by a variety of endogenous factors such as delirium, posttraumatic stress symptoms, depression, general anxiety, stress, inability to lie comfortably, pain, or by exogenous factors such as environmental noise, bright lighting, and repetitive staff interventions [8]. Poor sleep quality is highly correlated with various diseases, medical costs, as well as other indirect costs related to work absenteeism [1,9].

Good sleep quality is an indicator of wellbeing. Whereas; poor sleep quality results in increased co-morbidity, mortality, health care costs, and poor quality of life [10].Furthermore, poor sleep quality can cause an individual to feel tired the next day and may even be associated with long-term risks. Data in the literature has shown poor sleep quality to be frequently observed in hospitalized patients after surgery and is known to be associated with poor treatment outcomes. Many factors may impact poor sleep quality, and there is currently limited available data. Both short and long sleep durations have been associated with negative health outcomes in older and middle-aged adults [9]. Factors known to influence sleep quality includes; age, presence of comorbidities, body mass index, socioeconomic status, depression, anxiety, smoking, alcohol consumption, and physical activity, which should be taken into account [11,12].

Even if hospitalized patients often complain of sleep disturbances, still the severity and associated factors weren't stated. There are different studies on sleep quality in developed countries. But, no studies have been conducted on prevalence and predictors of sleep quality among adult patients after surgery and anesthesia in our study setting. To address the need for additional research regarding this important health problem, the present study was undertaken.

The present study aimed to determine the prevalence of poor sleep quality after surgery and its associated factors among adult patients in Amhara regional comprehensive referral Hospitals using the Pittsburgh sleep quality index assessment tool.

## 2 Methodology

### 2.1 Study design and setting

After taking ethical clearance from the ethical review committee of Debre Tabor University, a prospective multi-center cross-sectional study was conducted on 424 postsurgical patients who were selected by a systematic random sampling method. This study was already registered at www.researchregistry.com with a Research Registry UIN: researchregistry7403 and reported according to the STROCCSS criteria of 2021 [13].

**Study area and period**: This multicenter study was conducted at seven comprehensive referral hospitals. These areas were; Debre Tabor comprehensive referral hospital, Felege Hiwot comprehensive referral Hospital, Tibebe Gihon comprehensive referral hospital, Debre Markos comprehensive referral hospital, Dessie comprehensive referral hospital, Debre Birhan comprehensive Referral hospital, and University Of Gondar comprehensive referral hospital in Amhara regional state, Northern parts of Ethiopia from February 15/2021 to May 15/2021.

### 2.2 Study participants

The study participants were all eligible adult patients who were admitted to the post-surgical ward in Amhara regional comprehensive referral hospitals within the study period. The inclusion criteria were; all eligible volunteer adult patients undergoing any type of surgery. The exclusion criteria's were; a severe illness which required frequent vital sign monitoring, history of the previous admission within the study period, bedridden status, uremic encephalopathy, admission to an intensive care unit, a condition that can disturb the sleep-wake cycle including hepatic encephalopathy, Central nervous system infection, toxic and substance intoxication.

Written informed consent was taken from each study participant. A brief explanation and full disclosure of the benefit and risks of the study was done. They were also informed of their full right to refuse, withdraw, or completely reject part or all of their part in the study. Confidentiality was assured by removing identifiers and locking the questionnaires after data collection in a secured area.

### 2.3 Variables of the study

#### 2.3.1 Dependent variable

Postoperative poor Sleep quality.

**Independent variables:** Sociodemographic factors (sex, age BMI, occupation, income, marital status, educational status, residence, religion, total monthly income), Substance use, comorbidity, psychological factors (anxiety, depression, and pain), Surgical duration, type of surgery, Environmental factors (social support, Noise, light, pest) and anesthesia-related factors (type of anesthesia, duration of anesthesia, and type of drugs used perioperatively).

### 2.4 Operational definitions

**Poor sleep quality**- Based on the Pittsburgh sleep quality index tool, when the score is ≥ 6 points and developed short-term clinical effects of poor sleep quality, such as tiredness, fatigue, and loss of concentration [14].

**Good sleep quality**- When the Pittsburgh sleep quality index score is < 6 points.

### 2.5 Sample size determination & sampling procedure

There is no published study on the prevalence of poor sleep quality after surgery in the study area. So, the sample size of this study was calculated by using a single population proportion formula as follows.

$$n=\frac{{\left(\text{Z}\frac{\alpha }{2}\right)}^{2}\text{ p }\left(1-\text{p}\right)}{{\text{d}}^{2}}$$

where n = is the desired sample size; Z α/2 = is standard normal distribution usually set as 1.96 (corresponds to 95% confidence level); p = population proportion = 0.5), and d = degree of accuracy desired (marginal error is 5% (0.05).

$$So,\;n=\frac{{\left(1.96\right)}^{2}0.5\left(0.5\right)}{0.05^{2}},\;n=\frac{\left(3.8416\right)0.5\left(0.5\right)}{0.0025}$$

n=385

Then when we add 15% of the non-response rate, the final sample size is n = 58 + 385 = 443.

### 2.6 Sampling techniques

Using systematic random sampling techniques, all adult surgical patients who have undergone an operation at different operation rooms (Orthopedic, Gynecological, and major operation) of Amhara regional comprehensive referral hospitals were included during the collection of data in the study period.

## 3 Data collection procedures and tools for sleep quality

The questionnaires have different subsections; like sociodemographic factors, Quality of patient-related preoperative clinical factors, environmental-related factors, levels of social support, history of short term and long term substance use, anxiety and depression-related factors, intraoperative anesthesia and surgical related factors, postoperative related factors, and perioperative sleep-related factors were generally assessed using a PSQI self-administered questionnaire. Anxiety, depression, pain, and social support assessment tools were also included in the questionnaire.

Initially, the questionnaire was translated from a standardized English language to a standardized Amharic local language version. Using the Pittsburgh sleep quality index, we evaluated sleep habits 1 day before the operation, after the first night of surgery, and before discharge. This self-report questionnaire consists of 19 questions with seven subcategories: sleep quality, latency, duration, and disturbance; habitual sleep efficiency; use of sleep medications, and daytime dysfunction. The methods of scoring of the PSQI parameter were based on a 0 to 3 scale, where 3 reflects the extremely negative response on the Likert scale. The sum of the scores from all seven subcategories produces a global score ranging from 0 to 21 with higher scores associated with a poorer quality of sleep. The score distinguishing good from poor sleepers, using a PSQI of ≥6 points is indicated as sleep disturbance [15]. The PSQI is shown to have a higher degree of internal consistency with Cronbach's alpha of 0.85 and has been validated for clinical and laboratory diagnoses of good from poor sleep [16]. As different studies showed a global score greater than five found a specificity of 84.4% and a sensitivity of 98.7% as a marker for sleep disturbances in insomnia patients [15,17].

The hospital depression and anxiety scale were used to assess depression and anxiety. It has been validated in Ethiopia. It has two subscales: the anxiety subscale and the depression subscale. Each subscale contains seven items, giving a total of 14 items. It has a cut-off point ≥8 for each subscale suggestive of depression and anxiety [18].

Social support was assessed by Oslo social support scale measurements. It has 3 items which are classified as poor social support (3–8 score), intermediate (9–11 score), and strong social support (12–14 score [19].

**Postoperative pain:** It was assessed by using a numerical rating scale. The pain level was scored as no to mild pain (NRS<4) and moderate to severe pain (NRS ≥4) [20].

### 3.1 Data quality control

Pretest was done on 22 (5%) of patients. The data collectors were 7 BSC Nurses who were working in the ward during the study period. Training about the questionnaire was given for data collectors before the data collection period. The collected data was checked for its completeness and clarity on daily basis and corrections were made accordingly. Follow-up and supervision were done by the principal investigator throughout the study.

### 3.2 Data analysis and interpretation

First, the raw data was checked and entered into SPSS version 25 for analysis. For descriptive statistical measurements; frequency, percentages, median and interquartile range were used. For categorical data, a chi-square test was done. Bivariable and multivariable analyses were performed to determine whether each of the independent variables was associated with the outcome variable. Narrative expression, tables, and graphs were used to report the findings of the research. From all contributing factors, only variables with a p-value less than 0.2 during bivariable analysis were entered into the multivariable analysis. The strength of the association was presented by crude and adjusted odds ratio and 95% Confidence interval.

## 4 Result

A total of 443 adult surgical patients were included in this study based on the inclusion criteria with a 95.7% response rate. The data were collected from seven Amhara regional comprehensive referral hospitals. Nineteen patients were excluded from analysis due to incomplete data.

### 4.1 Sociodemographic characteristics of the study participants

The median and interquartile ranges for both age and body mass index of the study participants were 43(27–72) and 24.3(18.4–25) respectively. 240(56.6%) patients were males in gender. Regarding patients' BMI, 42.5% lays between the ranges of 18.5–24.9 kg/m^2^ (see Table 1).

**Table 1:** Shows the sociodemographic characteristics of study participants in ARRHs.

Sociodemographic factors	Category	Frequency (n)	Percentage (%)
Gender	Male	240	56.6
	Female	184	43.4
Religion	Muslim	118	27.8
	Orthodox	206	48.5
	Protestant	76	17.9
	Others	24	5.6
Age	18–24 yrs	23	5.4
	25–54 yrs	154	36.3
	55–64 yrs	152	35.8
	>64 yrs	95	22.4
Educational status	Illiterate	86	20.3
	Elementary school	167	39.4
	Secondary school	90	21.2
	College and above	81	19.1
Occupation	Employed	79	18.6
	Merchant	126	29.7
	Student	15	3.5
	Labor worker	14	3.3
	Housewife	73	17.2
	Unemployed	117	27.6
Marital status	Single	90	21.2
	Married	167	39.4
	Divorced	90	21.2
	Widowed	77	18.2
Monthly Income	<2500 ETB	367	86.6
	>2500 ETB	57	13.4
Residency	Urban	180	42.5
	**Rural**	**244**	**57.5**

### 4.2 Clinical and other patient-related factors

Among the study participants, 323 (76.2%) of patients were ASA I, 75 (17.6%) patients were ASA II, 20(4.72%) were ASA III, and the remaining were categorized under ASA IV cases. Hypertension was the most common comorbidity diagnosed in 40 (39%) of patients. Whereas; asthma, diabetes mellitus, and congested heart failure were diagnosed in 23 (23.3%), 18 (18%), and 20 (19.7%), respectively. Anxiety has occurred in 43.6%. Whereas, depression developed in 23.6% of adult post-surgical patients (see Table 2).

**Table 2:** shows patient-related variables and their distribution in Amhara regional comprehensive Referral Hospitals (n = 424).

Variables	Category	Frequency n(%)
Anxiety	Yes	185 (43.6%)
	No	239 (56.4%)
Depression	Yes	100 (23.6%)
	No	324 (76.4%)
Pain level	No to mild pain	45 (10.6%)
	Moderate to severe pain	379 (89.4%)
Social support	Poor	158 (37.3%)
	Moderate	126 (29.7%)
	Good	140 (33%)
History of Substance use	Yes	43 (10.1%)
	No	381 (89.9%)
Type of anesthesia	General anesthesia	257 (60.6%)
	Regional anesthesia	167 (39.4%)
Type of surgery	Elective	159 (37.5%)
	Emergency	265 (62.5%)
Comorbidity	Yes	65 (15.3%)
	No	359 (84.7%)

### 4.3 Prevalence of sleep quality

**Fig. 1:**
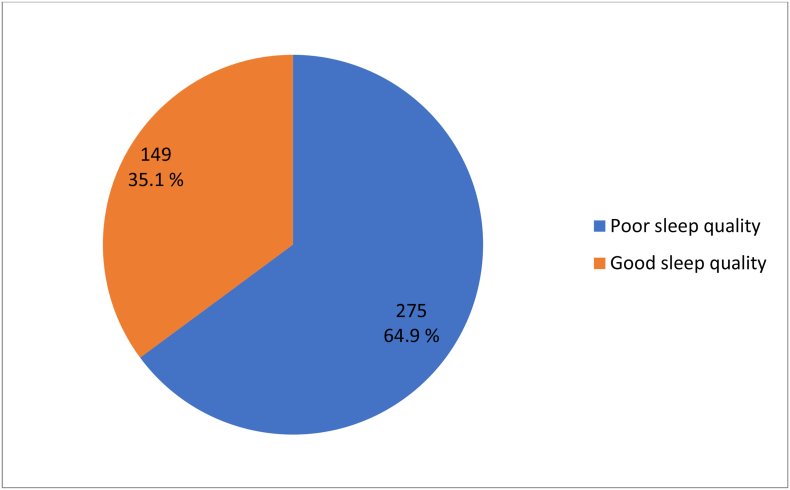
Shows the prevalence of sleep quality in comprehensive referral hospitals.

Using the PSQI assessment tool, among the study participant the prevalence of poor sleep quality was 64.9% (95% CI: 60.4, 69.3) (See Fig. 1).

### 4.4 Associated factors of poor sleep quality

Using the bivariable and multivariable logistic regression analysis, variables which had shown association with poor postoperative sleep quality were age range 25–54 years (AOR = 15.13; (95% CI: 1.76, 130.8)), male gender (AOR = 4.81; (95% CI: 1.11, 20.8)), educational level of secondary school (AOR = 6.29; (95% CI: 1.21, 32.8)), patient monthly income less than 2500 birr (AOR = 3.77; (95% CI: 1.86, 7.62)), anxiety (AOR = 2.53; (95% CI: 1.35, 4.73)), depression(AOR = 22.8; (95% CI: 6.94, 75.60)), light exposure(AOR = 19.60; (95% CI: 1.75, 207.40)), poor social support (AOR = 1.98; (95% CI: 1.08,3.61)), being emergency surgery (AOR = 2.46; (95% CI: 1.41, 4.30)) and having history of moderate to severe postoperative pain (AOR = 38.18; (95% CI: 6.21, 234.97)) (see Table 3).

**Table 3:** Shows Bivariable and multivariable logistic regression analysis results of factors associated with poor sleep quality among postoperative adult surgical patients in ARRHs.

Variables	Category	Poor sleep quality		COR (95% CI)	AOR (95% CI)	P - value
		Yes	No			
Age (years)	18–24	10(3.6%)	13(8.7%)	1	1	
	25–54	105(38.2%)	49(32.9%)	2.78(1.14,6.79)	15.2(1.76, 130.8)	0.013
	55–64	98(35.6%)	54(36.2%)	2.35(0.97,5.73)	0.65(0.171, 2.50)	0.537
	>64	62(22.5%)	33(22.1%)	2.44(0.96,6.16)	3.01(0.174, 53.3)	0.453
Gender	Male	161(58.5%)	79(53%)	1.25(0.83,1.86)	4.81(1.11, 20.80)	0.035
	Female	114(41.5%)	70(47%)	1	1	
Educational level	Illiterate	52(18.9%)	34(22.8%)	1	1	
	Read & write	107(38.9%)	60(40.3%)	1.16(0.68,1.99)	0.05(0.02, 1.84)	0.109
	Secondary school	60(21.8%)	30(20.1%)	1.30(0.70,2.42)	0.07(0.003, 1.64)	0.097
	College & above	56(20.4%)	25(16.8%)	1.46(0.77,1.78)	6.29(1.21, 32.80)	0.029
Marital status	Single	60(21.8%)	30(20.1%)	1	1	
	Married	108(39.3%)	59(39.6%)	1.05(0.56,1.95)	2.82(0.562, 14.16)	0.208
	Widowed	90(21.2%)	31(20.8%)	0.96(0.56,1.64)	0.47(0.028, 7.74)	0.595
	Divorced	48(17.5%)	29(19.5%)	0.87(0.46,1.63)	7.35(0.35, 6.25)	0.381
Income	<2500 ETB	247(89.8%)	120(80.5%)	2.13(1.21,3.74)	3.77(1.86, 7.62)	<0.001
	>2500 ETB	28(10.2%)	29(19.5%)	1	1	
Light exposure	Yes	96(34.9%)	32(21.5%)	0.51(0.32, 0.81)	19.1(1.75, 207.40)	0.015
	No	179(65.1%)	117(78.5%)	1	1	
Sound disturbance	Yes	97(35.3%)	36(24.2%)	1.71(1.09, 2.68)	8.55(0.83, 88.27)	0.072
	No	178(64.7%)	113(75.8%)	1	1	
Anxiety	Yes	133(48.4%)	52(34.9%)	1.75(1.16, 2.64)	2.53(1.35, 4.73)	0.004
	No	142(51.6%)	97(65.1%)	1	1	
Depression	Yes	90(32.7%)	10(6.7%)	6.76(3.39, 13.5)	22.9(6.94, 75.60)	<0.001
	No	185(67.3%)	139(93.3%)	1	1	
Hx of lifetime substance use	Yes	42(15.3%)	14(9.4%)	1.74(0.92,3.30)	2.53(0.92, 6.94)	0.072
	No	233(84.7%)	135(90.6%)	1	1	
Social support	Good	79(28.7%)	61(40.9%)	1	1	
	Moderate	80(29.1%)	46(30.9%)	1.34(0.82,2.19)	0.79(0.44, 1.46)	0.459
	Poor	116(42.2%)	42(28.2%)	2.13(1.31,3.47)	1.98(1.08, 3.61)	0.027
Postoperative pain level	No to mild	43(15.6%)	2(1.3%)	1	1	
	Moderate to severe	379(84.4%)	147(98.7%)	0.07(0.018,0.31)	38.2(6.21, 234.90)	<0.001
Type of surgery	Emergency	162(58.9%)	103(69.1%)	1.56(1.02,2.38)	2.46(1.41, 4.30)	0.002
	Elective	113(41.1%)	46(30.9%)	1	1	

## 5 Discussion

The purpose of this study was to assess postoperative poor sleep quality among adult patients in Amhara regional comprehensive referral hospitals. Again it was also aimed to see their association with sociodemographic factors, preoperative and intraoperative factors as a predictor of postoperative sleep quality.

Based on the finding of this study, the overall prevalence of postoperative poor sleep quality was 64.9% (95% CI: 60.4, 69.3). Accordingly, there was a poor postoperative sleep quality among adult surgical patients in Amhara regional comprehensive referral hospitals.

The present study found a similar and consistent result with a study done in Amanuel mental specialized hospital among epileptic patients, which was 65.4%, in Chinese 67.3% and Malaysia 61% [5,21,22].

Different studies previously revealed that postoperative sleep disturbance was still high and was inadequately managed in different areas of the world with an incidence between 16% and 67.3%. The prevalence of poor sleep quality in this study was higher compared with a study done in Hawassa referral hospital among HIV patients, which was 57.6%, and in Saudi Arabia, 55.4% [10,23]. This difference might be due to having a larger sample size, and sociodemographic differences of the study participants.

This study result showed that adult surgical patients with ages between 25 and 54 years were 15.2 times more likely to develop postoperative sleep disturbance compared with those extreme ages. The present study was supported by a report from the USA, which showed that the odds of developing the postoperative poor quality of sleep was 0.96 times higher compared with those older age patients (95% CI 0.93–0.99, p = 0.004) [24]. The possible justification could be due to the high risk of stress, exposure to substance use, and drug addiction in these age group patients.

At the same time, the present study also revealed that patients who developed moderate to severe postoperative surgical pain were more likely to develop a poor quality of sleep compared with those patients' pain status of no to mild state. This report is supported by studies done in Pennsylvania and Maryland, USA, which showed that a significant association of pain with postoperative poor sleep quality [[25], [26], [27]]. This is due to the effects of untreated postoperative pain which results in a disturbance of our physiologic and psychological activities [28].

This study result also reported that surgical patients with educational status of college and above levels were 6.29 times more likely to develop the postoperative poor quality of sleep compared with those who had educational levels of secondary school and below. The result of this study was supported by a report done in Saudi Arabia which showed a strong association of poor sleep quality with the academic performance of students in higher education academies [29]. The reason could be due to having extra activity in clinical practice, which exerts heavy stress on students and demands long hours of study [30].

Similarly, this study revealed that patients who had low monthly income were 3.77 times more likely to develop poor postoperative quality of sleep compared with those patients having an adequate income. This outcome was supported by a study done in Norway, and the USA which showed that there was a strong association between the poor quality of sleep and patients with below poverty threshold income with an adjusted odds ratio of 3.5 and 2.81 respectively [31,32]. The possible justification could be due to an increase in stress among poor individuals and a lack of guarantee for their day-to-day socioeconomic and health insurance demand [33].

On the other hand; the present study revealed that male gender patients were 4.81 times more likely to develop the poor postoperative quality of sleep compared with those female surgical patients. This study report was supported by a study done in South Korea [34] and the USA showed that being male gender had a strong association with poor postoperative quality of sleep [35]. The possible reason might be due to the exposure of male patients to addictive substances like smoking and alcohol, which will disturb the patient's mode of sleep [36].

In addition; this study result showed that postoperative surgical patients who were anxious and depressed were 2.53 and 22.9 times more likely to develop a poor quality of sleep compared with those who were non-anxious and non-depressed individuals, respectively. This report was supported by a study done in Japan which showed that the risks of developing poor sleep quality among depressed and anxious individuals were odd ratio [OR]: 1.09, 95% CI: 1.03–1.15) and (OR: 1.17, 95% CI: 1.11–1.24) respectively [37]. The possible justification might be the negative effects of anxiety and depression on the normal functions of the central nervous system which will end up with sleep disturbance [38,39].

At the same time; the present study result revealed that postoperative surgical patients who were done under emergency surgery were 2.46 times more likely to develop a poor quality of sleep compared with those who were done under elective schedules. This report was supported by a study done in Australia which showed that the risk of developing poor sleep quality was increased among emergency post-surgical patients compared to elective surgical cases [40]. The possible reason for this report could be due to hemodynamic instability among emergency surgical cases that contributed to the poor postoperative quality of sleep [41].

On the other hand, those individuals who had poor social support were 1.98 times more likely to develop poor sleep quality compared with those surgical patients who had moderate and good social support from their family or society. This report is supported by a study done in the USA that showed that patients who had poor social support were 1.24 times more likely to develop poor postoperative quality of sleep compared with those individuals who had good social support [42]. The possible justification could be social support to reduce negative emotions such as stress and anxiety. It also improves self-efficacy and share empathy [43].

Lastly; this study result revealed that postoperative patients who were exposed to light radiation in their waiting room were 19.1 times more likely to develop a poor quality of sleep compared with those patients who had lived in dark rooms. This report was supported by a study done in Japan that showed that the odds of developing the poor quality of postoperative sleep were 2.9 times higher among light exposed individuals than those non-exposed patients [44]. The possible justification could be the supersensitive nature of some patients to nocturnal melatonin suppression by light compared with healthy individuals [45].

## 6 Conclusion

Poor sleep quality among adult post-surgical patients was significantly high. Based on the present study; male gender, patient age between 25 and 54 years, educational status college and above, having a history of anxiety and depression, poor social support, low monthly income, light exposure, being emergency surgery, and history of having moderate to severe pain had a significant association with poor sleep quality. Therefore; Clinicians need to prioritize postoperative sleep quality assessment and needs to minimize factors inducing postoperative sleep disturbances.

## Strength of the study

This study was the first regarding postoperative sleep quality in Ethiopia and it gave the insight to do further research. At the same time, this study was multi-center, which includes seven comprehensive referral hospitals.

## Limitation of the study

The limitation of this study were the heterogeneity of study participants and the lack of a longer follow-up period.

## Recommendation of this study

Based on this study, the prevalence of poor sleep quality was high. So, we recommend for clinicians to assess sleep quality in the postoperative period and to manage the associated factors appropriately. And also, we recommend for interested researchers to do a further study regarding the quality of postoperative sleep with a high-level study design and with a longer follow-up period. Our recommendations also extend for hospital managers to allocate trained manpower and resources.

## Funding

None

## Ethical approval

Ethical approval was gotten from Debre tabor University.

## Sources of funding

None.

## Author statement

We have contributed for the preparation and development of whole contents of this manuscript.

SS initiated the idea, Collected data, carried out the study, and involved in drafting the manuscript. He also critically reviewed the manuscript for intellectual content. EF Contributed to preparation of the manuscript. Both authors are also contributed in critically revision of the paper and provided the final version.

## Consent

Consent was taken from individual patients.

## Registration of research studies

1. Name of the registry: Research registry.com.
2. Uniduque Identifying number or registration ID: 7403.
3. Hyperlink to your specific registration (must be publicly accessible and will be checked):

## Guarantor

Shimelis Seid Tegegne.

## Provenance and peer review

Not commissioned and externally peer-reviewed.

## Declaration of competing interest

The authors declare that there were no conflicts of interest.

## Acronym and abbreviations

ASA: American Society of Anesthesiologist
AOR: Adjusted Odd Ratio
ARRH: Amhara regional comprehensive Referral Hospital
BMI: Body Mass Index
CI: Confidence Interval
COR: Crude Odd Ratio
NRS: Numerical Rating Scale
PQSI: Pittsburgh Quality Sleep Index
SPSS: Statistical package for social science
